# QTL analysis to identify genes involved in the trade-off between silk protein synthesis and larva-pupa transition in silkworms

**DOI:** 10.1186/s12711-024-00937-z

**Published:** 2024-09-30

**Authors:** Rui Gao, Chunlin Li, Ang Zhou, Xiachao Wang, Kupeng Lu, Weidong Zuo, Hai Hu, Minjin Han, Xiaoling Tong, Fangyin Dai

**Affiliations:** State Key Laboratory of Resource Insects, Key Laboratory of Sericultural Biology and Genetic Breeding, Ministry of Agriculture and Rural Affairs, College of Biotechnology, Yibin Academy of Southwest University, Southwest University, Chongqing, 400715 China

## Abstract

**Background:**

Insect-based food and feed are increasingly attracting attention. As a domesticated insect, the silkworm (*Bombyx mori*) has a highly nutritious pupa that can be easily raised in large quantities through large-scale farming, making it a highly promising source of food. The ratio of pupa to cocoon (RPC) refers to the proportion of the weight of the cocoon that is attributed to pupae, and is of significant value for edible utilization, as a higher RPC means a higher ratio of conversion of mulberry leaves to pupa. In silkworm production, there is a trade-off between RPC and cocoon shell ratiao(CSR), which refers the ratio of silk protein to the entire cocoon, during metamorphosis process. Understanding the genetic basis of this balance is crucial for breeding edible strains with a high RPC and further advancing its use as feed.

**Results:**

Using QTL-seq, we identified a quantitative trait locus (QTL) for the balance between RPC and CSR that is located on chromosome 11 and covers a 9,773,115-bp region. This locus is an artificial selection hot spot that contains ten non-overlapping genomic regions under selection that were involved in the domestication and genetic breeding processes. These regions include 17 genes, nine of which are highly expressed in the silk gland, which is a vital component in the trade-off between RPC and CSR. These genes are annotate with function related with epigenetic modifications and the regulation of DNA replication et al. We identified one and two single nucleotide polymorphisms (SNPs) in the exons of teh *KWMTBOMO06541* and *KWMTBOMO06485* genes that result in amino acid changes in the protein domains. These SNPs have been strongly selected for during the domestication process. The *KWMTBOMO06485* gene encodes the *Bombyx mori* (Bm) tRNA methyltransferase (BmDnmt2) and its knockout results in a significant change in the trade-off between CSR and RPC in both sexes.

**Conclusions:**

Taken together, our results contribute to a better understanding of the genetic basis of RPC and CSR. The identified QTL and genes that affect RPC can be used for marker-assisted and genomic selection of silkworm strains with a high RPC. This will further enhance the production efficiency of silkworms and of closely-related insects for edible and feed purposes.

**Supplementary Information:**

The online version contains supplementary material available at 10.1186/s12711-024-00937-z.

## Background

As the global population continues to grow, providing enough food becomes increasingly challenging. Recently, there is growing interest in insects as a highly promising new food and feed source because they have been shown to contain substantial amounts of protein, unsaturated fatty acids, high levels of minerals, and various vitamins [1]. Around 1900 insect species are consumed by humans worldwide [2] and those that can be easily reared can serve as a low-cost and high-value source of feed. Their widespread application to livestock feed is of great significance to reduce feed costs (which account for 70% of total production costs) and enhance livestock production efficiency. The silkworm was one of the first domesticated insects and is reared in many countries, including China and India, to produce high-quality silk. In addition, the silkworm pupa has long been consumed for food, as the pupa has a very high nutritional value, with proteins providing 18 amino acids. Eight of these essential amino acids fulfil the WHO/FAO recommendations [3, 4]. Silkworm pupae are also used as a feed ingredient for animals such as poultry and fish [5, 6]. Furthermore, as silkworms have been domesticated and under selection for thousands of years, they can be easily reared indoors and on a large-scale to produce pupae. Therefore, expanding their use beyond silk production to the food and feed industry is of significant importance and breeding specialized silkworm strains is fundamental to food and feed applications.

For edible silkworm breeding, the ideal strain should have a high pupa weight (PW) and, in particular, a high pupa to cocoon ratio (RPC), which depends on a high conversion rate from mulberry leaves to pupa. The domesticated silkworm belongs to the Lepidoptera. After five instars of growth, mature larvae spin cocoons and the remaining parts undergo metamorphosis to become pupae. Traits that are closely related to silk protein synthesis and larval-pupal transformation include cocoon weight (CW), cocoon shell weight (CSW), pupal weight (PW), CSR, and RPC. CSR and RPC represent the proportion of the weight of the entire cocoon that is attributed to silk preotein and pupae, respectively. CSR and RPC are complementary to each other and their values represent the relative efficiency of silk protein synthesis and pupal weight gain during larval-pupal transformation. CSR and RPC have a complete negative correlation and are controlled by the same or tightly linked loci. Identifying the genes that control RPC and CSR is essential to understand the mechanisms underlying the trade-off between silk protein synthesis and larval-pupal transformation in silkworm metamorphosis, and is also an important basis for molecular breeding to cultivate edible silkworm strains with high PW and RPC.

For a long time, silk production has been the only breeding objective in sericulture. Thus, the focus of genetic research on silkworms has been on traits related to silk production, such as CSW and CSR. However, due to the complementary relationship between RPC and CSR, research on CSR can also contribute to unravel the genetic basis of RPC. Classic genetic analysis has shown that the heritability of CSR is high, with a broad sense heritability (h^2^_B_) > 85% and a narrow sense heritability (h^2^_N_) > 65% [7, 8]. CSR is a quantitative trait controlled by many minor quantitative trait loci (QTL) [9], which makes their identification challenging. Nevertheless, several studies have reported the detection of QTL for CSR [10, 11] but the fine mapping and identification of the genes that control RPC and CSR have been elusive.

In the current study, a segregating population was analysed by whole-genome resequencing to identify QTL and genes for RPC and CSR. These will enhance our understanding of the genetic basis of RPC and provide prospective loci breeding of silkworm strains with a high production efficiency for both food and feed purposes based on marker-assisted or genomic selection.

## Methods

A flowchart outlining the pipeline used for the identification and analysis of QTL for CSR and RPC is shown in Additional file 1: Fig. S1.

### Construction of the population and phenotype investigation

To map QTL for CSR and RPC, we first selected two silkworm strains (IS-Dazao and 872B) with significant trait differences, as the parents to construct the genetic segregating population. IS-Dazao has a higher RPC (and a lower CSR) and is a common laboratory strain with a reference genome sequence, while 872B is an improved strain with a lower RPC (and a higher CSR). IS-Dazao and 872B were preserved by self-crossing (female moths were randomly crossed with males from the same strain to produce the next generation) for more than 20 generations at the Silkworm Gene Bank of Southwest University. IS-Dazao females were hybridized with 872B males to generate an F_1_ population (IS-Dazao × 872B). Then, we randomly selected 12 IS-Dazao females and backcrossed them with 12 randomly selected F_1_ males, to obtain the BC_1_M (IS-Dazao♀ × (IS-Dazao × 872B) ♂) population, including 12 moth areas (individuals from one female moth) of silkworm eggs. They were independently reared by moth area in the same feeding environment. The feeding temperature was 27 °C for the first to third instars and 25 °C for the fourth and fifth instars, with a 12:12 h (L:D) photoperiod.

Before analyzing the traits related to silk protein synthesis and larval-pupal conversion rate in the parental F_1_ and BC_1_M populations, the cocoons of each individual were opened to distinguish the sexes based on the pupal size and the features of their abdomen, at the eye coloring stage. Then, cocoon weight (CW) and cocoon shell weight (CSW) were recorded and the cocoon shell ratio [CSR (CSW/CW)], the pupa weight [PW (CW − CSW)], and the pupa-cocoon ratio [RPC (PW/CW)] were calculated.

### Sample bulking and whole-genome resequencing

#### Preparation of DNA pools

Sex has a significant effect on CSR and RPC. Thus, to remove the effect of sex, we chose only males for bulk segregant analysis (BSA). We focused the subsequent analysis on CSR only, because CSR and RPC are complementary. We chose the pupae with the bottom and top 10% from each moth area, to form two pools (CSR_low and CSR_high), with each pool including 160 individuals. Then, DNA was extracted and quantified for each pupa and DNA from each pupa in the CSR_low and CSR_high groups was mixed in equal amounts, to construct two CSR differential genome bulks [CSR_low bulk (CSR_L) and CSR_high bulk (CSR_H)].

#### High-depth DNA resequencing

The sample DNA were passed quality control checks, including DNA concentration, purity, and integrity, and re-sequenced by Beijing Novogene Bioinformatics Technology Co., Ltd. (Beijing, China). First, DNA was randomly fragmented into 350-bp fragments using a Covaris sonicator, then the TruSeq Library Construction Kit was used to construct genome libraries using the reagents and consumables recommended in the user manual, as follows: the DNA fragments were subjected to end repair and addition of poly-A tails; then, sequencing adapters were ligated to the DNA fragments and PCR amplification was performed after further purification and length selection to complete the entire library preparation. Sequencing was performed on an Illumina HiSeq 2500 platform.

### Sequencing data quality control and variant calling

The original sequencing data were first subjected to the following quality controls: (1) removal of paired-end reads with adapters; (2) if the proportion of Ns in a single-end sequencing read exceeded 10% of the read length, the corresponding paired-end reads were removed to ensure data quality; (3) if the proportion of low-quality bases (Q ≤ 5) in a single-end sequencing read exceeded 50% of the read length, the corresponding paired-end reads were removed.

The resulting clean reads were aligned to the reference genome from SilkBase v2.1(http://silkbase.ab.a.u-tokyo.ac.jp/cgi-bin/index.cgi) [12] using the BWA-MEM algorithm version 0.7.10 (http://bio-bwa.sourceforge.net) with the “mem -t 4 -k 32 -M” parameters. Subsequent processing, including read sorting and removal of duplicates, was performed using the SAMTOOLS program version 1.14 (http://www.htslib.org/) [13] and the Picard software version 2.26.6 (http://broadinstitute.github.io/picard/) [14].

The Genome Analysis Toolkit (GATK) version 4.2.3.0 (https://gatk.broadinstitute.org/) [15] was used for realignment with the “IndelRealigner” parameter and variants (including SNPs) and insertion-deletions (Indels) were called with the “HaplotypeCaller” parameter, according to GATK best practice and recommendations. For SNPs in each bulk, the allele (reference and alternate) read depths were also calculated. The “CombineGVCFs” parameter was used to combine the variant genotypes between CSR_L and CSR_H. The SNPs and Indels of the combined file were then extracted with the “SelectVariants” parameter. SNPs were further filtered by applying the following cutoffs: QUAL < 30; MQRankSum < -12.5; FS > 60.0; ReadPosRankSum < -8.0; MQ < 40.0; QD < 2.0; SOR > 3.0; and DP < 8.0.

### Data import, filtering, and QTL mapping

To map the QTL for CSR, we conducted BSA using the next-generation sequencing data and two methods (QTL-seq [16] and G' analyses [17]), with the R Package (QTLseqr version 0.7.5.2; https://github.com/yuanlizhanshi/QTLseqr) [18], according to the main pipeline from the software developer recommendations, as briefly described in the following.

First, we used the “VariantsToTable” parameter of GATK4 to transform the above-filtered SNPs into table format and specified the SNPs as the QTLseqr input. Next, the SNPs were filtered according to the allele read depth (the number of all reads covering the alleles of a SNP obtained by sequencing) in the two bulks, with the “refAlleleFreq = 0.10, minTotalDepth = 100, maxTotalDepth = 500, depthDifference = 100, minSampleDepth = 40, and minGQ = 99” parameter with:

1 $$Ref \,allele \,frequency=\frac{Ref \,allele \,depth_{CSR\_H}+Ref \,allele \,depth_{CSR\_L}}{Total \,read \,depth \,for \,both \,bulks}.$$

In Eq. (1), *Ref allele depth*_*CSR_H*_ and *Ref allele depth*_*CSR_L*_ represent the read depth of the reference alleles in the two gene bulks, CSR_H and CSR_L, respectively; Total read depth for both bulks represent the total read depth of reference and alternate alleles in both CSR_H and CSR_L; *Ref allele frequency* represent the ratio of the read depth of reference alleles in the two bulks to the total read depth.

QTL-seq is the most commonly used method for BSA, which was developed by Takagi et al. [16]. In this method, the $\Delta (SNP-index)$ for each SNP was defined as the difference of the low value bulk *SNP-index* from the high value bulk *SNP-index*. The relevant formula are as follows:

2 $$SNP- index\,_{per\, bulk}=\frac{Alternate\, allele \,depth}{Total\, read \,depth}$$

3 $$\Delta \left(SNP-index\right)=(SNP-index)_{CSR_-H}-(SNP-index)_{CSR_-L}.$$

In Eq. (2), *SNP-index* in both high value bulk and low value bulk, defined as the proportion of the read depth of the alternate allele at each SNP, relative to the total read depth, after mapping the sequencing reads to the reference genome; In Eq. (3), $\Delta (SNP-index)$ represent the difference of the *SNP-index* of CSR_L from the CSR_H.

The larger the absolute value of $\Delta (SNP-index)$, the greater the difference in allele depth between the two DNA pools, and the greater the difference in the contribution from the two parental genomes, indicating a stronger linkage between the SNP and QTL that cause phenotypic differences between the two segregating groups. By averaging the $\Delta (SNP-index)$ over a sliding window, and through a simulated statistics, which repeatedly simulating the allele depths of each SNP in the specified population and calculating the $\Delta (SNP-index)$, for 10,000 times, the 95th and 99th percentile values of all simulation results are ultimately calculated to estimate the confidence intervals threshold [16]. The regions in which $\Delta (SNP-index)$ exceeded the confidence interval threshold (CI_95, CI_99) were identified as QTL for CSR.

G' analysis is an alternate method to evaluate statistical significance of QTL from next-generation sequencing (NGS) based BSA was proposed by Magwene et al.[17]. G' analysis calculates the G statistic (Eq. (4)) for each SNP based on observed and expected allele depths and then uses a sliding window with a tricube smoothing kernel to obtain the smoothed G' statistic. The relevant formula are as follows:

4 $$G=2*\sum {n}_{i}*ln\frac{obs\left({n}_{i}\right)}{\text{exp}\left({n}_{i}\right)}.$$

5 $$exp\left({n}_{1}\right)=\left({n}_{1}+{n}_{2}\right)*\frac{{n}_{1}+{n}_{3}}{{n}_{1}+{n}_{2}+{n}_{3}+{n}_{4}}, exp\left({n}_{2}\right)=\left({n}_{2}+{n}_{1}\right)*\frac{{n}_{2}+{n}_{4}}{{n}_{1}+{n}_{2}+{n}_{3}+{n}_{4}}, exp\left({n}_{3}\right)=\left({n}_{3}+{n}_{1}\right)*\frac{{n}_{3}+{n}_{4}}{{n}_{1}+{n}_{2}+{n}_{3}+{n}_{4}}, exp({n}_{4})=({n}_{4}+{n}_{2})*({n}_{4}+{n}_{3})/({n}_{1}+{n}_{2}+{n}_{3}+{n}_{4}),$$

In Eq. (4), ${n}_{i}$ are ${n}_{1}$, ${n}_{2}$, ${n}_{3}$ and ${n}_{4}$, as in Eq. (5). With ${n}_{1}$ and ${n}_{2}$ represent the reference allele depth and ${n}_{3}$ and ${n}_{4}$ the alternate allele depth for the CSR_H and CSR_L gene bulks, respectively. $obs({n}_{i})$ and $exp({n}_{i})$ represents the observed and expected allele depths, respectively, where the former is the actual alleles depth obtained by NGS of the CSR_H and CSR_L gene bulks, and the later is defined by Eq. (5).

In the G' method, *p*-values, which is derived from the G' value, and genome-wide Benjamini-Hochberg false discovery rate (FDR) [19] adjusted *p*-values are calculated for each SNP. The region with a − log_10_(*p*-value) above the FDR of 0.01 were defined as QTL for CSR.

The QTL-seq and G' methods were performed using the two main analysis functions of QTLseqr [16–18] (runQTLseqAnalysis and runGprimeAnalysis). The mapping results were plotted using the function plotQTLStats, the significant regions are extracted by getSigRegions, and a QTL summary was output using getQTLTable.

### Analysis of selective sweeps

The SNPs of 51 Wild, 205 Local, and 194 Improved silkworm strains (see Additional file 2: Table S1) were extracted from re-sequencing data of 1082 samples published by our group [20] for the analysis of selective sweeps. Because the CHN-I and JPN-I are two kept Improved populations which underwent independent breeding processes, we divided the Improved strains into two groups, CHN-I (105 samples) and JPN-I (89 samples), and identified signatures of selection separately based on the sequence data from each group. After filtering out SNPs with a missing rate > 50% using the vcftools software package version 0.1.16 (https://github.com/vcftools/vcftools) [21], nucleotide diversity (π), interpopulation sequence divergence (*F*_ST_), and reduction of nucleotide diversity (ROD) of Wild vs. Local and Local vs. the two Improved groups were calculated using a sliding window approach with 5-kb windows in 500-bp steps [22]. Taking the top 1% as cut-off value for *F*_ST_ between Wild and Local groups and the lowest 5% as cut-off value for π_Local_, and further confirmed the selective signatures of domestication with the top 5% as cut-off value for ROD. Similarly, windows that exceeded the top 5% as cut-off value for *F*_ST_ between Local and Improved groups, the lowest 5% as cut-off values for π_CHN-I_ or π_JPN-I_, and the top 5% as cut-off value for ROD, were identified as the signatures of artificial selection in the breeding stage. Neighboring windows in the silkworm genome that displayed signatures of selection were integrated into a genomic segment, and finally all the genomic segments that were identified to have been under selected during domestication and during breeding were identified. Since a few or no SNPs contained in some windows, we manually deleted and filled the windows if required to correspond their π values in different groups and to cocalculate the ROD. The detailed procedure of the detection of signatures of selection and the manual correction is illustrated in Additional file 3: Fig. S2a.

### Variant annotation, gene prediction, and gene tissue expression patterns

The annotated database of the silkworm, *Bombyx mori*, was built with snpEff-5.0-0 (https://pcingola.github.io/SnpEff/) [23] using new *Bombyx mori* gene models (2017), using Silkbase 2.0 as the reference genome database. SnpSift-4.3.1t-2 (https://pcingola.github.io/SnpEff/ss_introduction/) [24] was used to extract the SNPs in the identified genomic regions with selection signatures. If the identified genomic segments contained SNPs that were located within a gene and/or in its regulatory regions, the predicted genes were considered as candidate genes; if the identified genomic segments contained SNPs that were located only in intergenic regions, the nearest predicted gene was selected as a candidate gene. Based on the full-length transcriptome data of KAIKObase version 4.0.0 (https://kaikobase.dna.affrc.go.jp/) [25], the average number of fragments per kilobase of transcript per million fragments mapped (FPKM) values were calculated from three biological replicates of each tissue, then the FPKM values of the selected genes were extracted, their expression pattern was analyzed, and a heatmap was drawn using the R pheatmap function. As silk gland is the organ where silk protein is synthesized, it is the vital determinator of CSR and RPC. Therefore, to identify candidate genes for further investigation, we defined the highly and specifily expressed genes in silk gland by considering two criteria simultaneously. One is that with average FPKM value > 10 in at least one section of the silk gland, which can be divided into three sections: the anterior silk gland (ASG), middle silk gland (MSG), and Posterior silk gland (PSG). The second criteria is that the sum of FPKM values in the silk gland (ASG + MSG + PSG) ≥ 20% in all tissues [25].

### Identification and analysis of allele frequency differences

#### Extraction of variants

SNPs, Indels, and structural variants (SVs) in the genomic and upstream 2-kb regulatory regions of the identified candidate genes were extracted using vcftools version 0.1.16 [21] in different groups of silkworms. Briefly, we first investigated the SNPs and Indels in 450 silkworm strains, including the 51 Wild, 205 Local, and 194 Improved strains. To fully investigate sequence variants, we further analyzed the SVs in the genomic regions where these genes are located in 39 Wild, 160 Local, and 113 Improved strains (see Additional file 2: Table S1) based on the pan-genome data published by our group [20]. Then the “filter (ANN [*]. IMPACT = 'HIGH') | (ANN [*]. IMPACT = 'MODERATE')” parameter of vcftools was used to extract variants that had the strongest impact on gene structure and biological functions.

#### Analysis of allele frequency differences

The allele frequencies (AF) of each variant that was predicted to have a strong impact in the three populations was calculated using the “vcftools -freq” parameter and the divergence in AF between Wild and Local, and Local and Improved groups, was analyzed using a Chi-square test using IBM SPSS Statistics version 28.0.1 (http://www.ibm.com/cn-zh/products/spss-statistics).

#### Protein domain prediction

To further investigate whether the variants with significant differences in AF between the populations were located in a particular domain of the protein encoded by the gene, we predicted the protein domains using the HMMER software package version 3.3.2 (http://www.ebi.ac.uk/tools/hmmer) based on the amino acid sequence of the proteins.

### CRISPR/Cas9 knockout

The CRISPR/Cas9 technology was carried out to knockout the candidate gene *BmDnmt2* [26]. Briefly, two sgRNAs were designed on the CRISPRdirect website (http://crispr.dbcls.jp/) [27] and the forward and reverse primers (see Additional file 4: Table S2) were designed and synthesized in The Beijing Genomics Institute (BGI) (China). After PCR amplification and purification, the products were transcribed into gRNA with the Transcript Aid T7 High Yield Transcription Kit (Thermo Scientific Company, Waltham, Massachusetts, USA). The purified gRNA was diluted 20 times and 2 × RNA loading buffer was added, heated for 10 min at 70 °C, and quickly placed on ice. Two percent agarose gel was used for electrophoresis to detect bands. Both qualified gRNAs and Cas9 protein (Invitrogen, Carlsbad, California, USA) were mixed (0.5:0.5 µg/µL), and incubated at 37 °C for 15 min, followed by microinjection into newly laid non-diapause IS-Dazao eggs (n = 320). We chose IS-Dazao as the wild-type strain because it is a conventional experimental strain, which has a high-quality reference genome and a good health and vitality.

### Screening and sequence detection of homozygous knockout lines

The 20 hatched injected eggs, comprising the G0 generation, were reared and 11 of them were grown to the moth stage when their wings were cut in half, placed in a 1.5-mL centrifuge tube and crushed after adding a lysis solution (DNAiso Reagent; TaKaRa, Kyoto, Japan). DNA was extracted using the DNAiso Reagent following the manufacturer’s instructions and purified with 75% absolute ethanol. DNA detection primers were designed on the sgRNAs genome flank for PCR amplification. Heterozygous individuals with double or more bands (n = 7) were self-bred to produce G1 offspring. One hundred and seventy-four G1 individuals were investigated, among which 21 transheterozygous individuals were identified. The PCR amplification product was ligated into the pEASY-Blunt Zero Cloning Kit (TransGen Biotech, Beijing, China) for cloning and positive spots were selected for sequencing. Individuals with the same mutation were self-bred to produce G2 offspring. Among the 168 investigated G2 individuals, 16 were with homozygous mutation, which were self-bred to produce G3 offspring. One moth-area of the G3 homozygous (*BmDnmt2*-KO line) individuals was selected in order to rear them simultaneously with the wild-type line under the same conditions. Silk protein is synthesized in the silk gland, which is the most important organ that affects CSR and RPC. Thus, the silk glands were dissected on the 3rd day of the 5th instar of the larvae and RNA was extracted with TransZol Up Plus RNA Kit (TransGen Biotech) and reverse-transcribed into cDNA using a PrimeScript RT reagent Kit (TaKaRa). *BmDnmt2* cDNA primers were designed to detect the deletion in the *BmDnmt2* gene in the *BmDnmt2*-KO line. The primers are listed in Additional file 4: Table S2, and the flowchart for the screening and sequence detection of homozygous knockout lines is illustrated in Additional file 5: Fig. S3.

### Statistical analyses

The normality test of the CSR for the BC_1_M population was performed with the Shapiro-Wilk normality test. We obtained a *W* closer to 1 and a *P* > 0.05, which indicated that the CSR and RPC of BC_1_M was consistent with a normal distribution.

Divergence in AF of the variants between populations was tested using a Chi-square test in the crosstab of the analysis module of IBM SPSS Statistics software version 28.0.1. A *P-*value < 0.05 indicated that the genotype frequencies at a site were significantly different between populations.

A difference in the cocoon silk protein synthesis indices between the *BmDnmt2*-KO line and wild-type line was determined using an unpaired two-sided Wilcoxon test.

## Results

### Construction of mapping population and genomic sequencing of pools

**Fig. 1:**
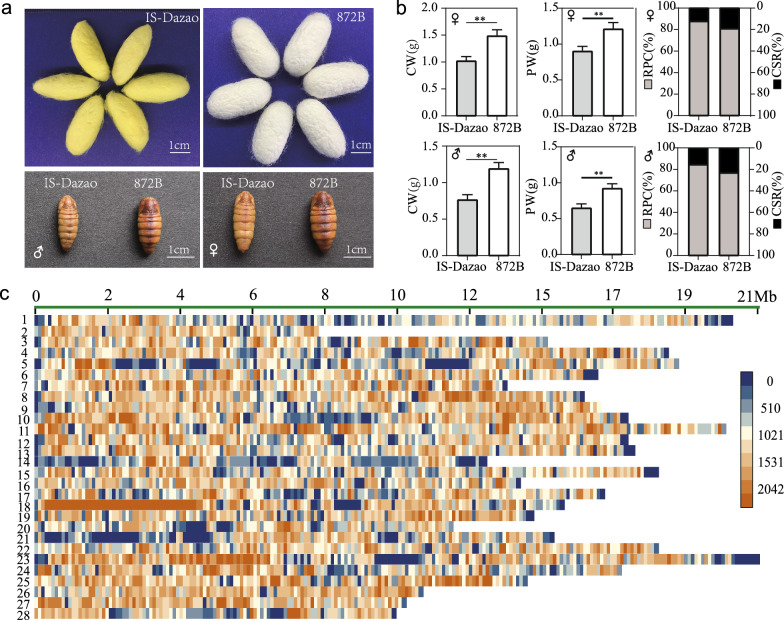
Trait performance for traits related to silk yield and pupa weight ratio in the two parental lines and the pooling sequencing experimental design of the bulk segregant analysis (BSA). **a** Photographs of the cocoon and pupa of the silkworm strains, IS-Dazao and 872B. **b** Cocoon weight (CW), pupa weight (PW), the ratio of pupa to cocoon (RPC) and cocoon shell ratio (CSR) of females and males from the IS-Dazao and 872B strains. Sample sizes *n* for IS-Dazao females and males are 80 and 90, respectively, and for 872B females and males of are 78 and 88, respectively. **c** SNP density map on each chromosome of the merged DNA pools of the CSR_L. and CSR_H. Y-axis: chromosome number; X-axis: physical distance of chromosomes; window size is 100 kb

To identify genes that control CSR and RPC, we selected IS-Dazao and 872B as parental strains, which exhibit significant differences for these two traits (Fig. 1a). IS-Dazao is a local strain that showed lower values for cocoon weight (average CW was 0.768 g for males and 1.021 g for females), cocoon shell weight (average CSW was 0.115 g for males and 0.118 g for females), pupal weight (average PW was 0.653 g for males and 0.903 g for females), and CSR (average value was 15.0% for males and 11.6% for females), but higher RPC (average value was 85.0% for males and 88.4% for females), with sample sizes of 80 and 90 for males and females, respectively. In contrast, the 872B strain displayed higher CW (average value was 1.200 g for males and 1.506 g for females), CSW (average value was 0.286 g for males and 0.29 g for females), PW (average value was 0.914 g for males and 1.216 g for females), and CSR (average value was 23.8% for males and 19.3% for females), but lower RPC (76.2% for males and 80.8% for females), with sample sizes of 78 and 88, for males and females, respectively (see Fig. 1b, Additional file 6: Fig. S4b and Additional file 7: Table S3). Since CSR and RPC are complementary, we focused the subsequent analyses only on CSR. Using IS-Dazao and 872B individuals as parents, we generated a backcross population. Among the 12 moth areas for BC_1_ individuals, on average 143 male individuals were present in each area, and the total population included 1714 males. CSR varied among moth areas, with a mean value ranging from 16.7 to 18.0%, and a large (maximum–minimum) range from 5.2 to 10.3% (see Additional file 6: Fig. S4a, c, and Additional file 8: Table S4). To ensure the accuracy of sampling, we selected individuals with 10% maximum and 10% minimum phenotypic values from each area to form the CSR_low and CSR_high sub-groups. Each sub-group contained 160 individuals and had an average CSR of 15.2% and 19.4%, respectively (see Additional file 6: Fig. S4d). Then, the genomic DNA of each individual was extracted and mixed in equal proportions to construct CSR extreme high (CSR_H) and low (CSR_L) gene pools.

To screen out the loci with significant differences in allele frequencies between the pools, we first re-sequenced the two gene pools at high depth. Genomic sequencing produced 188 Gb of clean data, with the CSR_L and CSR_H pools representing 88 Gb and 100 Gb, respectively. Q20 values (the error rate is less than 1 in 100 for each base call during DNA sequencing) of 97.95 and 97.85%, Q30 values (the error rate is less than 1 in 1000 for each base call during DNA sequencing) of 93.95 and 93.72%, and GC contents of 38.78 and 38.82%, were found for the CSR_L and CSR_H pools, respectively. Read mapping results showed that 99.16% and 99.12% of the clean reads for the CSR_L and CSR_H pools, respectively, mapped to the reference genome, and the average sequencing depth was up to 157.05 × and 174.25 × , respectively (see Additional file 9: Table S5). After variant calling, merging, and filtering, we finally obtained 5,987,733 SNPs and 1,313,988 Indels with high quality. The average number of SNPs per 100 kb was 1335 (Fig. 1c). The distribution of the SNPs showed that some regions were not covered by SNPs or had a low coverage. Further alignment and analysis showed that most of these regions were gaps or repeat-rich regions.

### QTL mapping and artificial selection analysis in candidate regions

To rapidly map QTL that control CSR and RPC, we calculated the *SNP-index* [16] of each pool based on the allele read depth in the two pools at each SNP site, then screened the CSR-associated SNPs. First, we filtered out 607,144 low-confidence SNPs, resulting in 4,209,531 SNPs on the 28 chromosomes. The SNP allele read depths were then statistically analyzed to check the quality of the data. The total read depth was mainly in the range of 250 to 400 × (see Additional file 10: Fig. S4a). The reference allele frequency was enriched to 0.75, which was consistent with the genetic characteristics of the BC_1_ population (see Additional file 10: Fig. S4b). In addition, the *SNP-index* of the CSR_H pool was normally distributed around 0.25, while the *SNP-index* of the CSR_L pool was normally distributed around a value lower than 0.25, and with a small peak at 0 (see Additional file 10: Fig. S4c, d), indicating the presence of the sites that are linked with CSR. These statistical results showed high confidence in the pre-sampling and SNP data.

**Fig. 2:**
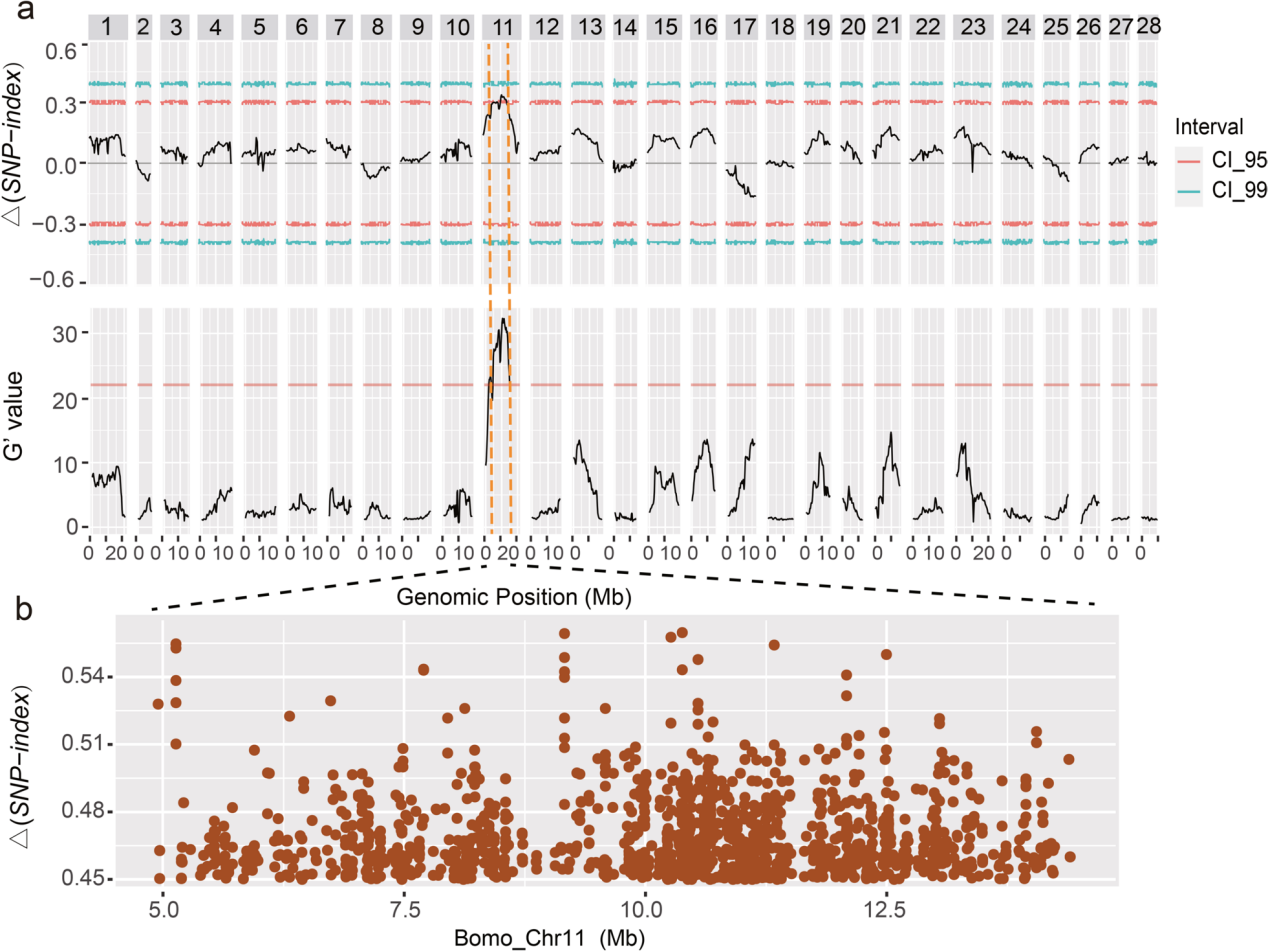
QTL mapping analysis of the CSR/RPC. **a** The Δ(*SNP-index*) and G' values of the SNPs in the merged CSR_L and CSR_H pools were calculated in the whole genome via a sliding window method with a window size of 1e^6^ bp. The numbers along the top of the figure represent silkworm chromosome numbers. X-axis: physical distance of each chromosome (Mb); the lines for CI_95 and CI_99 in Δ(*SNP-index*) means the two-sided confidence intervals of 0.95 and 0.99, respectively; the line in the G' value plot means FDR = 0.01. **b** Δ(*SNP-index*) statistics for each SNPs within the QTL region, and points indicate SNPs with a Δ(*SNP-index*) ≥ 0.45. X-axis: Physical position within the mapping region of silkworm Chr11

Based on SNP allele frequencies, we calculated the Δ(*SNP-index*) [16] and G' value [17] using a sliding window method [18], which showed that the mapping results of the two indices were relatively consistent and a QTL-controlling CSR was mapped to chromosome 11 [Chr11] (Fig. 2a). The region was located on *Bombyx mori* (Bomo)_Chr11 (between 4,799,421 and 14,572,536 bp, covering 9,773,115 bp). The region contained 72,313 SNPs, among which 8350 SNPs had a Δ(*SNP-index*) ≥ 0.4, and 1360 SNPs had a Δ(*SNP-index*) ≥ 0.45 (Fig. 2b), which suggests that this region is enriched in loci that are very strongly associated with to CSR.

**Fig. 3:**
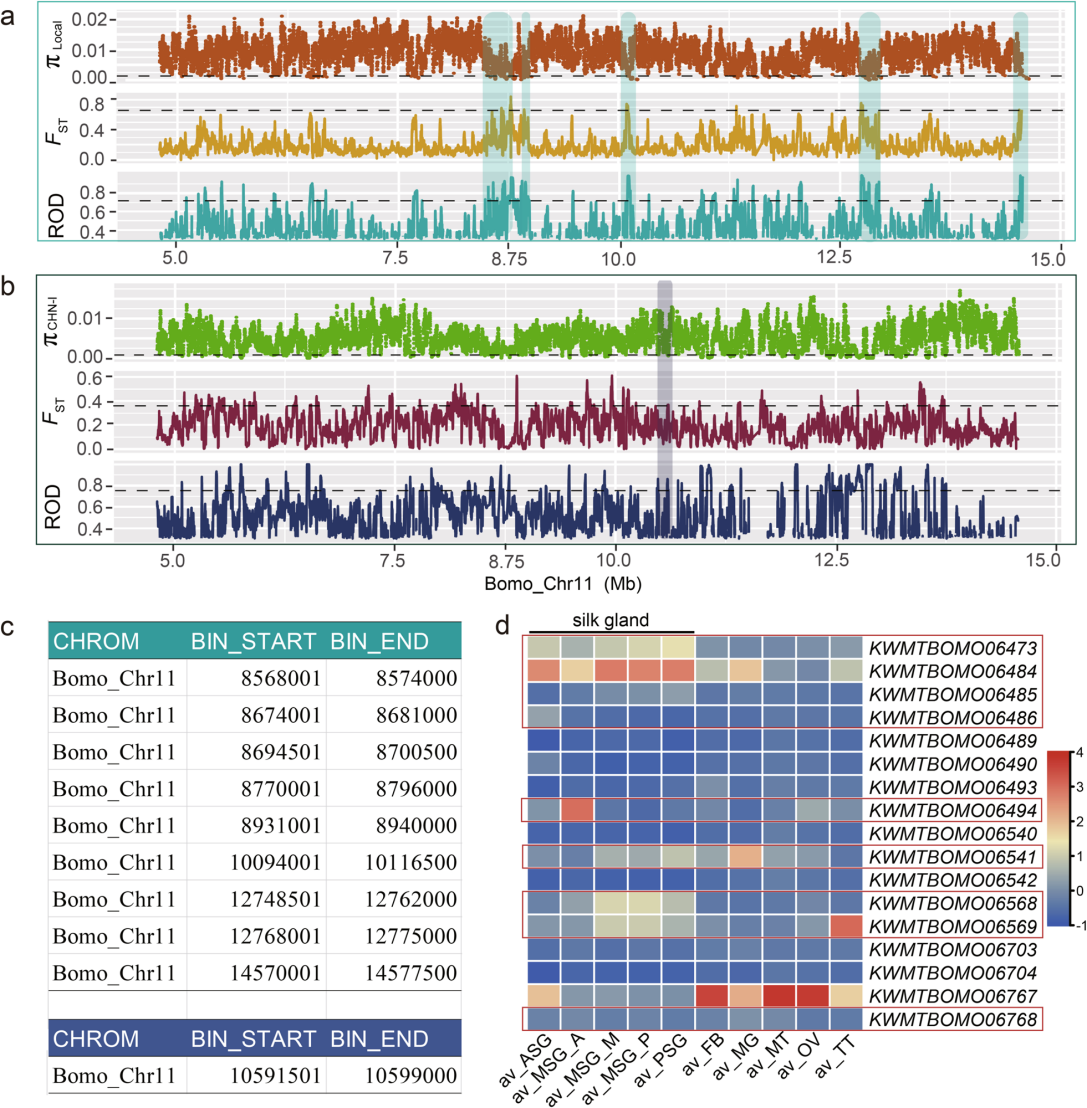
Selection signals within the mapped regions during domestication and genetic breeding processes. **a**, **b** Analysis of the selection pressure of the QTL region during domestication (**a**) and breeding (**b**) of silkworms via a sliding window method with a window size of 5 kb and a step-size of 500 bp. Threshold line in (**a**): π_Local_ ≤ lowest 5% (0.0014), *F*_ST Wild-Local_ ≥ top 1% (0.63) and ROD _Wild-Local_ ≥ top 5% (0.7). Threshold line in (**b**): π_CHN-I_ ≤ lowest 5% (0.0008), *F*_ST Local-CHN-I_ ≥ top5% (0.35) and ROD _Local-CHN-I_ ≥ top 5% (0.75). Green shadow box: region selected by domestication. Blue shadow box: region subjected to selection during CHN-I improvement. The SNPs are extracted from the re-sequenced data of silkworm strains in Additional file 2 Table S1, and sample sizes *n* of Wild, Local and CHN-I silkworms are 51, 205 and 105, respectively. **c** Genomic start and end positions of the window bins (bp) in the artificially-selected region. Up: domestication-selected regions. Down: improvement-selected region. **d** Heat-map of the tissue expression of genes in regions under artificial selection. Red boxes indicate the genes with a higher expression level in the silk gland (SG). ASG: anterior silk gland; MSG_A: anterior segment of middle silk gland; MSG_M: middle segment of middle silk gland; MSG_P: posterior segment of middle silk gland; PSG: posterior silk gland; FB: fat body; MG: midgut; MT: Markov tube; OV: ovary; TT: testis; av: mean FPKM values of three biological replicates. The data consisted of the FPKM values derived from the transcriptome of tissues dissected from the 3rd day of the 5th instar larvae of silkworm strain P50, as provided in KAIKObase version 4.0.0

Domestication and genetic breeding are two important processes that have selected for traits related to silk protein synthesis and, thus, have influenced CSR and RPC. To further identify the genes that control CSR and RPC in the region on Bomo_Chr11, detection of the significant artificial selection signals associated with domestication and genetic breeding processes was carried out. Nucleotide polymorphism (π), population differentiation index (*F*_ST_), and reduction of nucleotide diversity (ROD) were used to measure the strength of the selection signals. By meeting restrictive cut-offs, the QTL comprised nine genomic regions that were associated with strong domestication selection signals and one region that was strongly selected in the CHN-I strains (Fig. 3a–c and see Additional file 3: Fig. S2), indicating that this QTL was an artificial selection hot spot. Seventeen genes were located in or near the identified genomic regions and were defined as potential domestication and breeding-related genes (Table 1). The tissue expression profiles showed that nine of these 17 genes had a high and specialized expression pattern in the silk gland (Fig. 3d and see Additional file 11 Table S6), thus, they were selected as candidate genes that may be involved in the trade-off between cocoon weight and pupa weight, and were further investigated.

**Table 1:** Information on the genes with selection signals in the mapped region

Gene	Annotation	Location
*KWMTBOMO06473*	Protein arginine N-methyltransferase 7	Chr11(−):8,591,464–8,598,050
*KWMTBOMO06484*	Protein JTB-like	Chr11(+):8,673,211–8,674,900
*KWMTBOMO06485*	DNA cytosine-5 methyltransferase	Chr11(−):8,675,578–8,677,610
*KWMTBOMO06486*	Sphingosine kinase 1-like	Chr11(+):8,697,461–8,708,528
*KWMTBOMO06489*	Transposase	Chr11(−):8,755,028–8756180
*KWMTBOMO06490*	Cyclin-dependent kinase 14	Chr11(−):8,818,220–8885134
*KWMTBOMO06493*	85/88 kDa calcium-independent phospholipase A2	Chr11(+):8,929,015–8936000
*KWMTBOMO06494*	Protein cueball	Chr11(−):8,941,655–8,952,204
*KWMTBOMO06540*	Uncharacterized protein	Chr11(+):10,063,495–10087349
*KWMTBOMO06541*	Methionine sulfoxide reductase	Chr11(−):10,096,727–10106624
*KWMTBOMO06542*	Protein phosphatase 1 regulatory subunit 16A	Chr11(−):10,113,897–10152354
*KWMTBOMO06568*	DNA replication factor Cdt1	Chr11(−):10,587,083–10591464
*KWMTBOMO06569*	SRSF protein kinase 2	Chr11(+):10,591,805–10598290
*KWMTBOMO06703*	3-Phosphoinositide-dependent protein kinase 1	Chr11(−):12,734,520–12739740
*KWMTBOMO06704*	Endonuclease-reverse transcriptase	Chr11(+):12,798,730–12799819
*KWMTBOMO06767*	Extramacrochaetae protein	Chr11(−):14,499,424–14,503,681
*KWMTBOMO06768*	Uncharacterized protein	Chr11(+):14,578,572–14,579,700

### Analysis of sequence variants in the candidate genes

First, we analyzed the positional relationship between the nine candidate genes and the selection signals. The results showed that the selection signals were located in the intergenic region of the *KWMTBOMO06473* gene and within the flanking regulatory and coding regions for the other eight genes (Fig. 3c; Table 1), which suggests that these variants had important effects on these genes. Therefore, we scanned the full sequence of the nine genes for variants (including SNPs, Indels and SVs). The results showed that these genes harbored many sequence variants in the Wild, Local, and Improved silkworm populations (see Additional file 12: Fig. S6).

**Fig. 4:**
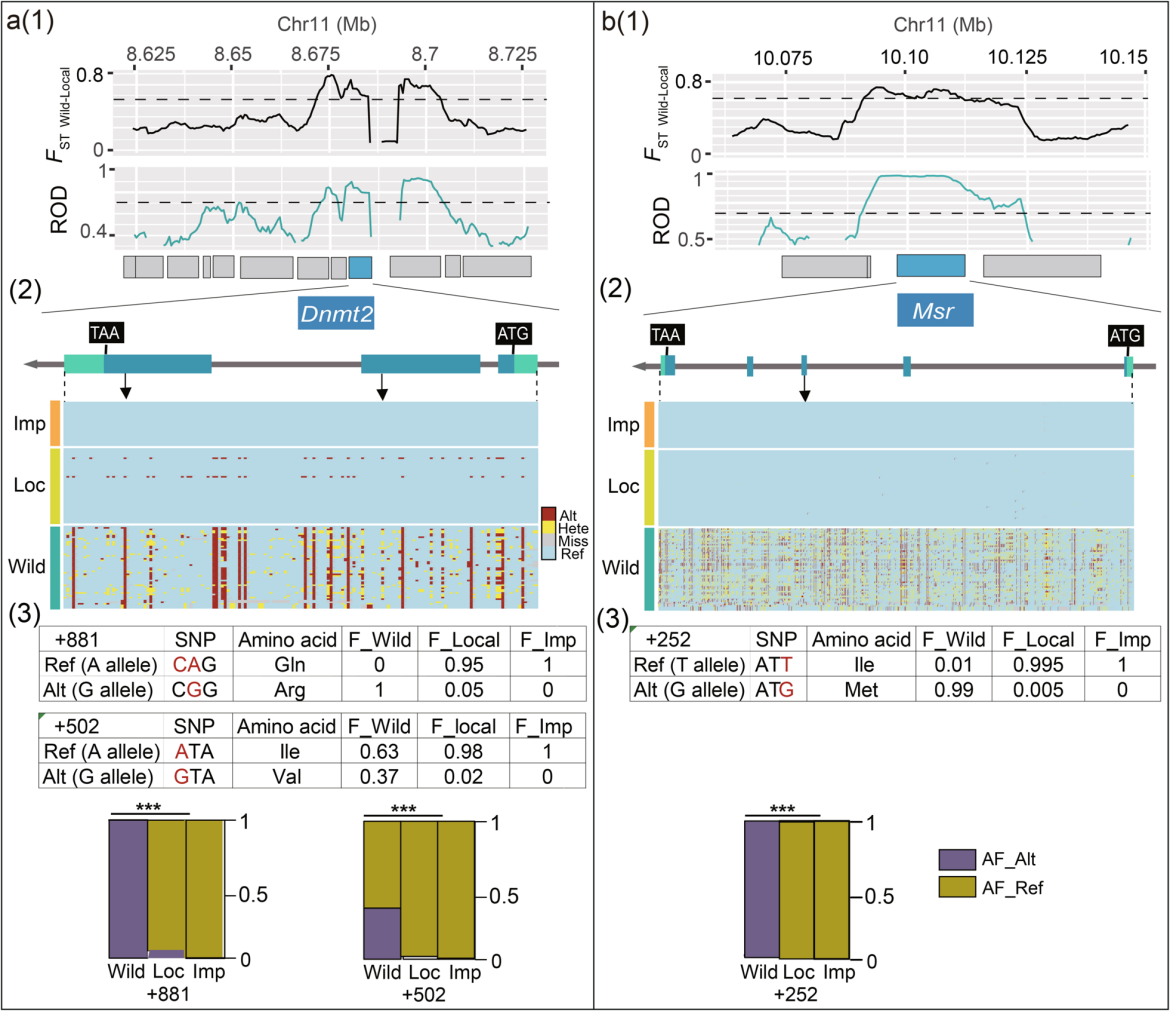
Sequence variation analysis of the candidate genes. **a1** Selection signal in the genomic region around the *BmDnmt2* gene during domestication of silkworm, with a sliding window size of 5 kb and a step-size of 500 bp. Threshold line: *F*_ST Wild-Local_ ≥ top 1% (0.62) and ROD _Wild-Local_ ≥ top 5% (0.7). **a2** The gray box indicates the predicted genes within the region. The blue box indicates the *BmDnmt2* gene, the combination of black boxes and lines is the gene structure diagram of *BmDnmt2*, and the bottom is the allele heat-map of SNPs in Wild, Local, and Improved populations in the genome region where *BmDnmt2* is located. The SNPs are extracted from the re-sequenced data of silkworm strains in Additional file 2 Table S1, Sample sizes *n* of Wild, Local and Improved silkworms are 51, 205 and 194, respectively. Blue: Reference allele, Red: Alternate allele, Yellow: heterozygous genotype, and Grey: missing genotype. Arrows indicate the SNPs that are located in the exons of the gene. From left-to-right are the + 881 and + 502 bases of the coding region, respectively. **a3** Allele frequency of SNPs in + 881 and + 502 bases of the coding region in different silkworm populations, and the corresponding amino acids encoded by each allele. Sample sizes of Wild, Local and Improved silkworms are the same with these in **a2**. The graphical description of the allele frequency is at the bottom. AF_Alt: allele frequency of the alternate allele of the SNPs in Wild, Local and Improved silkworms. AF_Ref: allele frequency of reference allele of the SNPs in the same groups. A Pearson chi-square test was used to compare the difference in allele frequencies between Wild and Local silkworm groups. ^***^The Chi-square value corresponded to a *P* < 0.001. **b** Selection signal in the genomic region around the *BmMsr* gene; legend details are the same as for Fig. 3a; the blue box indicates the *BmMsr* gene and the arrow indicates that the SNP is located at + 252 base of the gene coding region

Variants in the gene coding region directly affect the amino acid sequence of the protein, thereby change its function. Therefore, we first focused on the variants in the gene coding regions and investigated their allele frequencies in the three populations. The results showed that there was a large number of SNPs and SVs in the coding regions, but no Indel was detected. Thirteen SVs were detected in the exons of six genes, but there was no significant difference in allele frequencies between the populations (see Additional file 13: Table S7). One hundred and ninety-nine SNPs were detected in the exons of the eight genes that were under selection during domestication, among which seventeen SNPs were located in the exons of five genes that showed significant differences in allele frequencies between the two populations of the *Bombyx mandarina* and *Bombyx mori* Local strains [*P* < 0.05 (Chi-square test)], including *KWMTBOMO06473* (three SNPs), *KWMTBOMO06485* (two SNPs), *KWMTBOMO06486* (four SNPs), *KWMTBOMO06494* (seven SNPs), and *KWMTBOMO06541* (one SNP; see Additional file 14: Table S8). Further analysis revealed that only two SNPs in *KWMTBOMO06485* and one SNP in *KWMTBOMO06541* were located in the domains of the encoded proteins. Two mutations in the *KWMTBOMO06485* gene (the 502nd and 881st bases of the coding sequence) were located in the 2nd and 3rd exons of the gene, respectively (Fig. 4a). One mutation in the *KWMTBOMO06541* gene (the 252nd base of the coding sequence) was located in the 3rd exon (Fig. 4b). All three were missense mutations that resulted in amino acid changes in the resulting proteins, and their allele frequencies were significantly different or even several SNPs showed almost completely opposite alleles between the Wild and Local strains [*P* < 0.05 (Chi-square test); Fig. 4a, b]. In addition to those in the coding regions, variants located in the regulatory regions of the genes can also have significant regulatory effects on gene expression. By analyzing the variants located in the regulatory regions of the candidate genes, we identified a haplotype in the 5ʹ untranslated region (UTR) of *KWMTBOMO06568*, which consisted of five SNPs. The frequencies of the two genotypes of this haplotype changed significantly during the silkworm breeding progress, and were significantly associated with CSR and RPC in the germplasm resources of silkworms (see Additional file 15: Fig. S7). These findings suggest that the variants within the gene and its regulatory regions were strongly selected during the process of domestication and breeding, may have caused a change in gene function or expression, and contributed to an increase in CSR and a reduction in RPC in the target group.

### Functional validation of the role of *BmDnmt2* on CSR and RPC

**Fig. 5:**
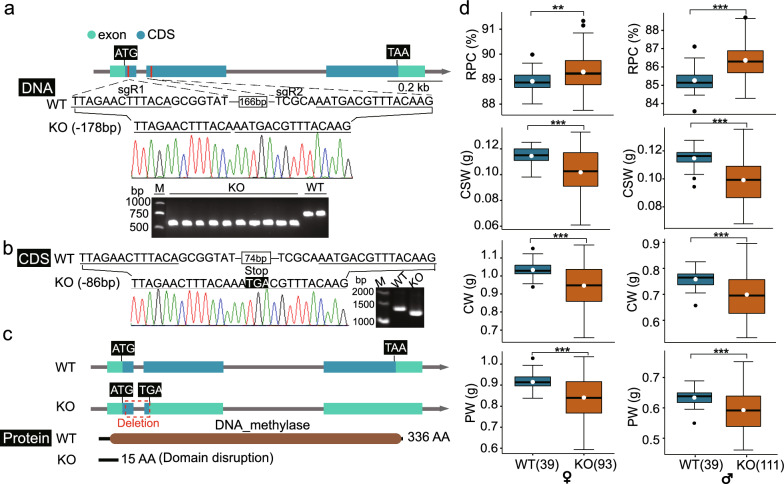
Effect of *BmDnmt2* on RPC and silk yield traits. **a** Detection of mutant alleles in the *BmDnmt2*-KO genomic sequence. The gene structure of *BmDnmt2* is shown at the top. The two sgRNA sites (sgR1 and sgR2) of *BmDnmt2* are marked in red, and the 178 bp deletion of *BmDnmt2*-KO between the two sites is shown in the middle. The sequencing results are shown below. At the bottom is the result of the PCR amplification of the deletion sequence in the wild-type and *BmDnmt2*-KO line. **b** Detection of mutant alleles in the *BmDnmt2*-KO coding sequence. There were 74 bp between the two sites in the wild-type, an 86 bp deletion in *BmDnmt2*-KO between the two sites in the coding sequence and a stop codon (TGA) was introduced. The results of base sequencing and gel electrophoresis are shown below. **c** Schematic diagram of structural changes in the *BmDnmt2*-KO protein. The brown box is the DNA methylase domain (PF00145.19). **d** Phenotypic investigation of RPC, CSW, CW, and PW in females (left) and males (right) of wild-type (WT) and *BmDnmt2*-KO (KO), respectively. Sample size is shown in parentheses following WT and KO, respectively. Center line, median; white dots, mean; box limits, upper and lower quartiles; whiskers, 1.5 × the interquartile range; black dots, outliers (**P < 0.01, ***P < 0.005, two-sided Wilcoxon test)

To confirm that the genes present in the hot spot affect CSR and RPC, we selected one candidate gene, *KWMTBOMO06485*, which encodes the tRNA methyltransferase, Dnmt2 of *Bombyx mori*, so it is also named as *BmDnmt2*, for further validation. Dnmt2 belongs to the DNA methyltransferase family and is involved in tRNA homeostasis, protein translation, and siRNA-mediated gene regulation. First, we used the CRISPR/Cas9 technology to knock out the *BmDnmt2* gene to study its role on CSR and RPC and other traits related to silk protein synthesis. Briefly, two sgRNAs were designed in the first and second exons of this gene, then it was knocked-out of from IS-Dazao (Fig. 5a). Compared with the wild-type, the *BmDnmt2* homozygous knockout line (*BmDnmt2*-KO) had a 178-bp deletion between the two sgRNAs (Fig. 5a and see Additional file 5: Fig. S3). Analysis of the coding sequence showed a deletion of 86 bp, which resulted in a frameshift variant and introduced an inter-frame stop codon between the first and second exons of *BmDnmt2* (Fig. 5b). Thus, the translated sequence contained only 15 amino acids, leading to a complete loss of function of Dnmt2 (Fig. 5c).

Then, we investigated differences in silk protein synthesis traits and larval-pupal conversion ratio between the wild-type and *BmDnmt2*-KO lines. The results showed a significant change of the trade-off between CSR and RPC in the *BmDnmt2* knock-out individuals of both sexes. In females, the average CSR value was 11.1 and 10.7% in the wild-type and *BmDnmt2*-KO lines, respectively, with a decrease of approximately 3.3%; the corresponding RPC showed a significant enhancement, with an average value of 88.9 and 89.3% in the wild-type and *BmDnmt2*-KO lines, respectively. Similar results were also obtained in males, with an average CSR value of 14.7 and 13.6% in the wild-type and *BmDnmt2*-KO lines, respectively, and a decrease of approximately 7.4%; the corresponding RPC showed a significant enhancement, with an average value of 85.3 and 86.4% in the wild-type and *BmDnmt2*-KO lines, respectively. [*P* < 0.01 or *P* < 0.001 (Wilcox test); Fig. 5d] and see (Additional file 16: Fig.S8). We also observed that the other related indices such as CSW, CW, and PW were significantly lower in the *BmDnmt2*-KO line than in the wild-type [*P* < 0.001 (Wilcox test); Fig. 5d]. The average CSW values decreased to a greater degree (approximately 11.1 and 14.3% in the *BmDnmt2*-KO females and males, respectively). This result shows that, in *Bombyx mori*, the *Dnmt2* gene may be involved in the trade-off between cocoon weight and pupa weight.

## Discussion

The domesticated silkworm has a long history of being consumed as food, but because the primary focus of silkworm breeding is on silk production, the main breeding objective has been on improving silk yield efficiency, which has limited research on increasing pupae productivity for edible and feed purposes. In this study, we identified a QTL for RPC, which has been under selection during domestication and selective breeding of silkworm. Within this QTL, a gene that controls RPC was identified by CRISPR/Cas9. This result lays an important foundation for the molecular breeding of high RPC silkworm strains.

### QTL for the trade-off between cocoon weight and *pupa* weight

Because CSR and RPC are complementary traits, which sum to one, the QTL that control CSR inevitably also influence RPC, thus regulating the balance between the two traits. Therefore, they can serve as dual targets for improving both CSR and RPC. In this study, we identified a QTL controlling CSR on Chr11, which was also an artificial selection hot spot of domestication and breeding. Our previous research also showed that this region carries a major locus for CSW [28, 29], which suggests that it is a hot spot region related to silk yield traits. Classic genetic analysis showed that CSR is a quantitative trait and QTL analysis identified multiple loci on different chromosomes, including 13, 18, and 19 [10, 11]. However, under our criteria, only one region controlling CSR was identified, on Chr11 (Fig. 2a). Detailed analysis of this QTL revealed two peaks, suggesting that it may consist of two closely-linked loci. However why was this QTL identified as a continuous region? Our explanation is that because we used a BC_1_ population for mapping analysis, in which fewer recombination events occurred during its formation, the mapping accuracy was low. We also detected SNPs that were closely associated with CSR on other chromosomes, including 1, 15, 16, 18, and 19. These SNPs were located one by one on the genome and several SNPs appear consecutively (N ≥ 4), which allows us to exclude the influence of sequencing errors to some extent. Therefore, these SNPs and their genomic regions may also be linked with CSR (see Additional file 17: Table S9). These results indicate that CSR is a quantitative trait controlled by multiple genes, and the relationship between the sequence variation, as well as genes in the above genomic regions and CSR are worthy of further study.

### The *BmDnmt2* gene affects the CSR and RPC

Knock-out of one of the candidate genes, *KWMTBOMO06485* (*BmDnmt2*), led to a significant increase in RPC and a decrease in CSR compared with the wild-type line. In addition, the knockout line showed a decrease for other related traits, including CSW, CW, and PW, which may be due to the high genetic correlations of CSR and RPC with other traits related to silk protein synthesis. Indeed, our previous study [30] showed high genetic correlations between traits related to silk protein synthesis, such as CSW, CSR, CW, and PW, with estimates ranging from 0.60 to 0.98. Another possible explanation is that, because the balance between CSR and RPC is a complex trait, which is directly related to the relative amount between CSW and PW, the effect of *BmDnmt2* on CSR/RPC may be the result of its effects on CSW and PW. Statistical analysis of the knockout line revealed that CSW decreased by 11 and 14% in males and females, respectively, while PW decreased by only 8 and 7% in males and females, respectively (Fig. 5d). This indicates that, although *BmDnmt2* affects both CSW and PW, the effect on CSW is greater, which suggests that *BmDnmt2* is involved in the trade-off between silk protein synthesis and larvae-pupa transition. In addition, the multiple effects of knocking *BmDnmt2* out also implies that this gene may play diverse roles in silk protein synthesis and even in silkworm development.

Dnmt2 was initially defined as a member of the DNA methyltransferase family because of the conservation of its catalytic motif, but later it was discovered to have a tRNA methylation activity [31]. Several reports have shown that it can catalyze m5C methylation of tRNA^Asp−GUC^, tRNA^Gly−GCC^, and tRNA^Val−AAC^ by targeting cytosine 38 (C38) residues [31, 32]. Methylation of these tRNAs by Dnmt2 have an essential role in protein translation. In mice, a double mutation in *Dnmt2* and *Nsun2*, the latter encoding the only other known cytidine-5 tRNA methyltransferase in mammals, results in the full loss of m5C in tRNAs, and reduces global protein translation [33]. Furthermore, *Dnmt2*‑knockout mice show a decreased ability to correctly distinguish near-cognate codons, resulting in reduced translation accuracy, and producing misfolded proteins and abnormal phenotypes [34]. Considering our findings on the function of *BmDnmt2* for RPC and CSR, as well as the crucial role of protein translation in silk protein synthesis and larval-pupal transition, we hypothesize that *BmDnmt2* may regulate the translation efficiency and accuracy of proteins which are required for silk protein synthesis and the growth of the silkworm, by catalyzing the methylation of several tRNAs. It is worth noting that one of the tRNA substrates of Dnmt2, tRNA^Gly−GCC^, is responsible for transporting glycine (Gly) to the elongated polypeptide chain, and Gly is one of the four major amino acids that constitutes silk protein. To validate this hypothesis, further investigations are needed, such as analysis of methylation differences between specific tRNAs, protein synthesis rates, and ribosome profiling to detect codon occupancy in the *BmDnmt2* knockout line. In addition to having a role in protein translation, *Dnmt2*-mediated tRNA methylation can also influence tRNA stability. Mutations in the *Dnmt2* gene in *Drosophila* lead to an increase in tRNA cleavage fragments, which are involved in the cross-talk with the siRNA pathway, thus regulating gene expression and resulting in reduced survival capabilities under stress conditions [35]. Moreover, mutations in *Dnmt2* can also impact the lifespan of *Drosophila* [36]. Therefore, *Dnmt2* may play a significant role in the regulation of larval development and affect RPC. It is interesting to note that *Dnmt2* is a multifunctional gene, as it has been reported to play roles in transposon silencing, RNA-mediated epigenetics, and cancer. Whether such functions of *Dnmt2* are related to its regulation on the trade-off between silk protein synthesis and larva-pupa transition is also worthy of further research.

### Other candidate genes for CSR and RPC

In addition to *BmDnmt2*, this hot spot region of artificial selection also includes other candidate genes. *KWMTBOMO06541* was identified as another gene with sequence variants in the protein domain region. It encodes a methionine-S-sulfoxide reductase (Msr). This enzyme catalyzes the reversible oxidation and reduction of methionine sulfoxide in proteins. It was reported to act as a downstream regulator of *FOXO* for prolonging the life span of organisms [37]. The health lifespan of silkworms, especially the duration of the mulberry feeding period of larvae, has an important effect on pupa weight, which suggests an association between RPC and Msr.

Another candidate gene, *KWMTBOMO06568*, possesses a haplotype of five SNPs in its 5'-untranslated region (UTR) and was identified to under selection during breeding. It encodes the DNA replication factor 1 (Cdt1), which collaborates with the origin recognition complex (ORC) and Cdc6 to load the replicative helicase MCM onto eukaryotic DNA replication origins, allowing replication initiation [38]. Dysregulation of this process can lead to incomplete DNA replication, cell cycle arrest, and potentially severe developmental abnormalities and diseases. As a crucial component and regulatory target of replication licensing, *Cdt1* has also been shown to be essential for endoreplication in *Drosophila* [39]. Considering that endoreplication in the silk gland cells provides a large amount of DNA for efficient silk protein synthesis, in *Bombyx mori*, *Cdt1* may also affect the endoreplication efficiency of silk gland cells, thereby influencing traits related to silk protein synthesis such as RPC and CSR. Moreover, in *Arabidopsis*, *Cdt1* was shown to be a key target for the coordination of cell proliferation, endoreplication in differentiation, and development [40]. Therefore, this gene also holds great potential as a target for regulating the trade-off between larval transformation into cocoons and pupa. Taken together, these results suggest that the identified artificial selection hot spot on Chr 11 comprises more than one gene that influence CSR and RPC.

## Conclusions

We have identified an artificial selection hot spot that is involved in the trade-off between silk protein synthesis and larvae-pupa transition during silkworm domestication and breeding. This hot spot region may comprise several genes that control CSR and RPC. As an example, we confirmed that the *Bombyx mori Dnmt2* gene present in this region is a candidate gene for traits related to RPC and CSR. The genes identified in this study can serve as targets involved in the trade-off between the efficiency of silk protein synthesis and larvae-pupa transition, and high-RPC or CSR breeding strains can be created through transgenic techniques or gene editing. Furthermore, associated SNPs can be used as molecular markers for marker-assisted breeding or serve as foreground markers for genomic selection in bi-directional breeding aimed at enhancing CSR or RPC.

## Supplementary Information

Additional file 1: Figure S1. Title: Flow of the process of the identification and analysis of the QTL controlling CSR and RPC throughout the paper.

Additional file 2: Table S1. Title: Information of Wild, Local, and Improved (CHN-I and JPN-I) silkworm groups.

Additional file 3: Figure S2. Title: Detailed procedure for the analysis of selective sweeps and the signatures of JPN-I Improvement. Description: a. Detailed procedure for the analysis of selective sweeps. b. π of JPN-I; *F*_ST_ and ROD between local and JPN-I silkworm groups in the QTL region on Chr11.

Additional file 4: Table S2. Title: Primers used in this study.

Additional file 5: Figure S3. Title: The flowchart of screening and sequence detection of homozygous knockout lines.

Additional file 6: Figure S4. Title: Descriptive statistics of the BC_1_M population and pooling sequencing experimental design of the bulk segregant analysis (BSA). Description: (a) Method used for the production of the BC_1_M population. (b and c) CSR of males from the IS-Dazao, 872B, F_1_ and each BC_1_M moth area, with the abscissa representing the serial number of each moth area in the BC_1_M population (1-12). Sample sizes n = 79, 62, 50 and 1,714, respectively. (d) Frequency distribution of CSR in the BC_1_M population. Shapiro-Wilk normality test (W = 0.97154; *P* < 2.2e-16). The left and right images represent the mean CSR for the two pools (CSR_L and CSR_H); n represents the number of individuals per pool.

Additional file 7: Table S3. Title: Analysis of silk yield in the parental and F_1_ cross populations.

Additional file 8: Table S4. Title: Analysis of CSR in the BC_1_M backcross populations.

Additional file 9: Table S5. Title: Quality of the genome resequencing data of CSR_L and CSR_H.

Additional file 10: Figure S5. Title: Frequency distribution of read depth and allele frequency of CSR_L and CSR_H SNPs. Description: (a) Total read depth in two gene pools. (b) Frequency of the reference allele in the two gene pools. C. *SNP-index* of CSR_H. D. *SNP-index* of CSR_L.

Additional file 11: Table S6. Title: FPKM values of the tissue expression of genes in/near the genome regions under artificial selection.

Additional file 12: Figure S6. Title: Detection of genomic sequence variations in the candidate genes for CSR.

Additional file 13: Table S7. Title: Strutural variants in the coding region of the candidate genes and their allele frequencies in silkworm populations.

Additional file 14: Table S8. Title: SNPs in the coding region of the candidate genes and their allele frequencies in silkworm populations.

Additional file 15: Figure S7. Title: Haplotype in the 5' UTR of *BmCdt1*, with the two genotypes that are associated with CSR and CSW between Local and Improved silkworm populations.

Additional file 16: Figure S8. Title: Phenotypic investigation of CSR in females (left) and males (right) of the wild-type (WT) and *BmDnmt2*-KO (KO) lines.

Additional file 17: Table S9. Title: SNPs closely linked with CSR (with Δ(*SNP-index*) ≥ 0.4), which were consecutively appeared along the genome (N ≥  4).

## Data Availability

The datasets supporting the conclusions of this study are included within the article and as additional files.
