# Supplementary figures and images for: QTL analysis to identify genes involved in the trade-off between silk protein synthesis and larva-pupa transition in silkworms
Source: Genet Sel Evol. 2024 Sep 30;56:68. doi: 10.1186/s12711-024-00937-z (PMC11440889; doi:10.1186/s12711-024-00937-z)

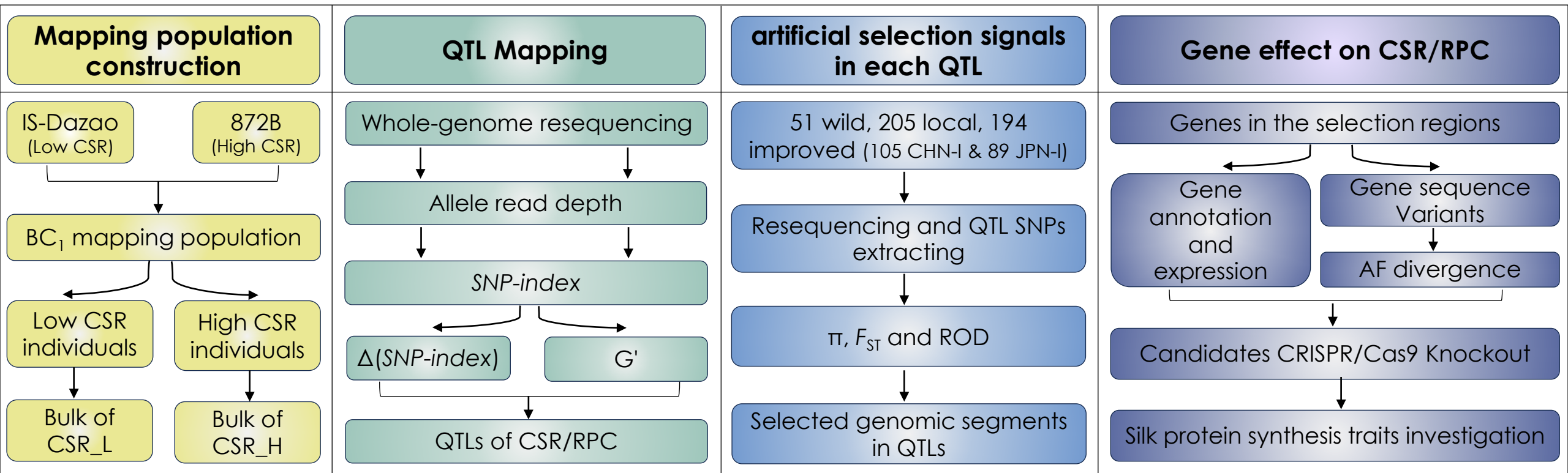

Supplement: Supplementary file 1 — Additional file 1: Figure S1. Title: Flow of the process of the identification and analysis of the QTL controlling CSR and RPC throughout the paper. [file 12711_2024_937_MOESM1_ESM.pdf]

a

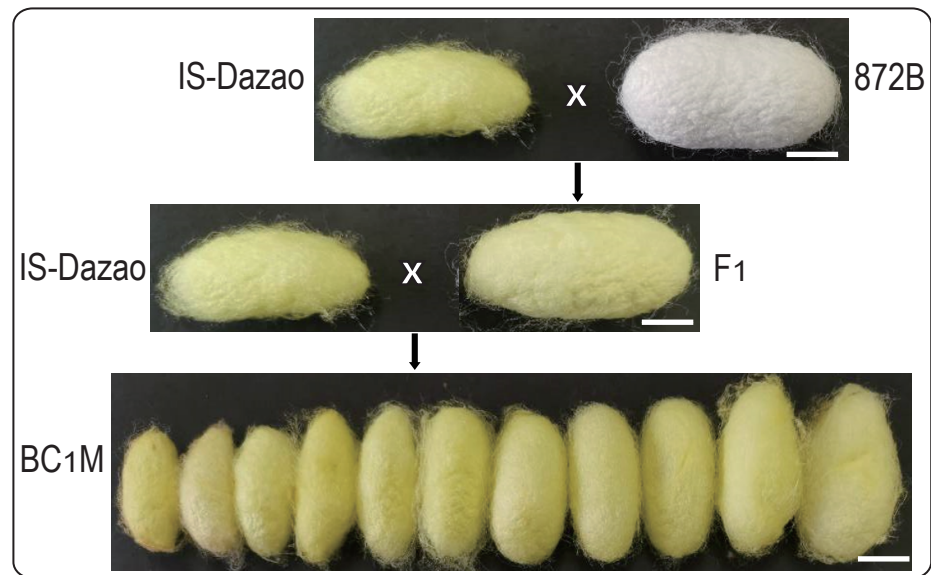

b

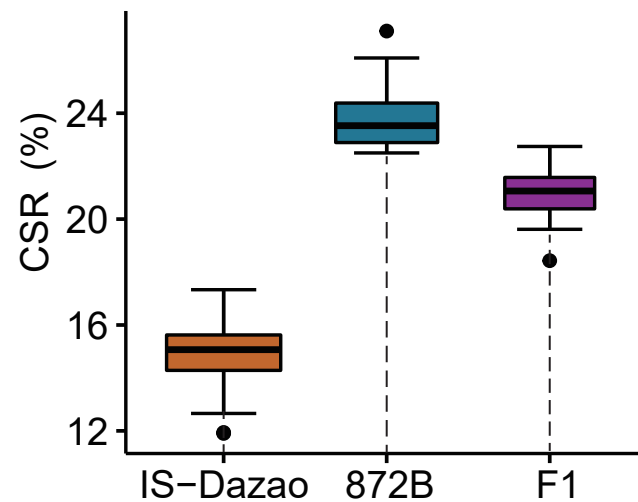

c

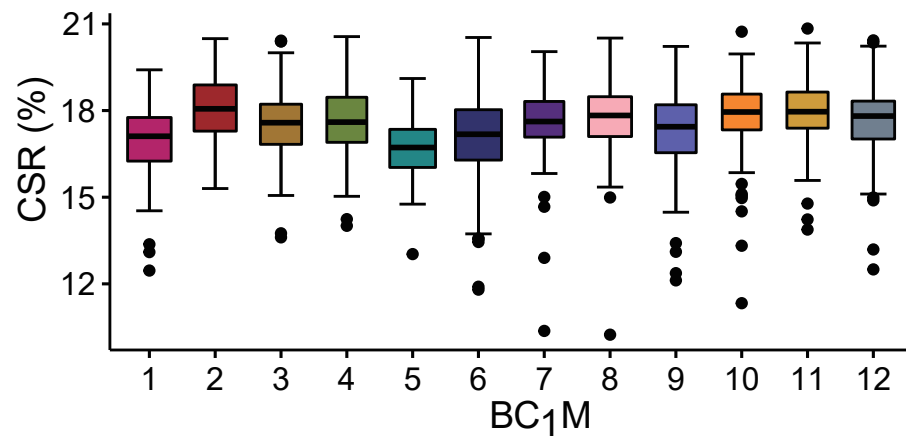

d

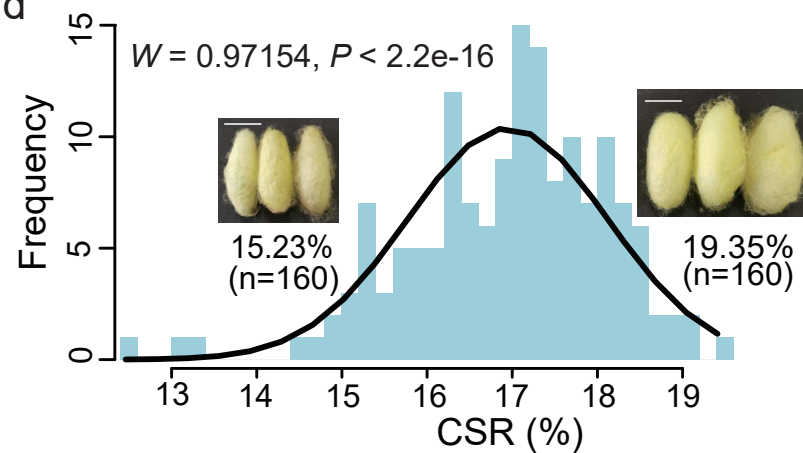

Supplement: Supplementary file 6 — Additional file 6: Figure S4. Title: Descriptive statistics of the BC1M population and pooling sequencing experimental design of the bulk segregant analysis (BSA). Description: (a) Method used for the production of the BC1M population. (b and c) CSR of males from the IS-Dazao, 872B, F1 and each BC1M moth area, with the abscissa representing the serial number of each moth area in the BC1M population (1-12). Sample sizes n = 79, 62, 50 and 1,714, respectively. (d) Frequency distribution of CSR in the BC1M population. Shapiro-Wilk normality test (W = 0.97154; P < 2.2e-16). The left and right images represent the mean CSR for the two pools (CSR_L and CSR_H); n represents the number of individuals per pool. [file 12711_2024_937_MOESM6_ESM.pdf]

a

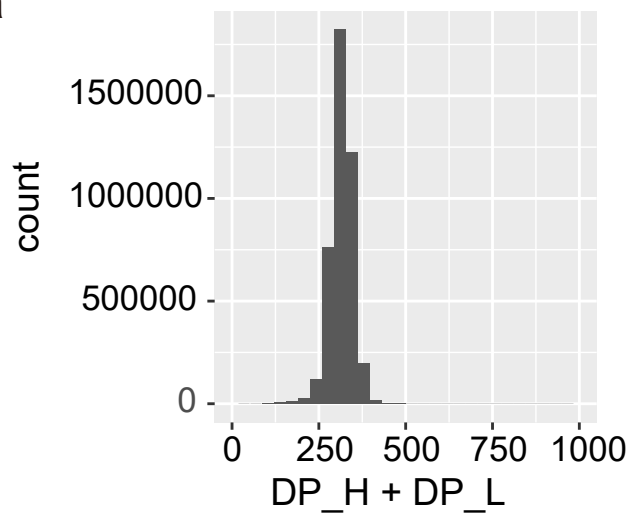

b

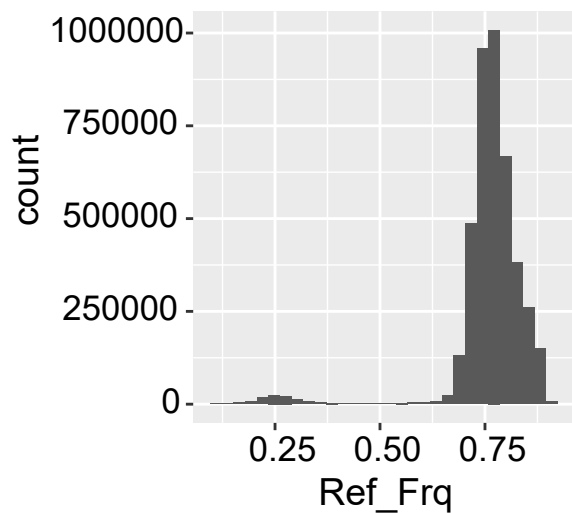

c

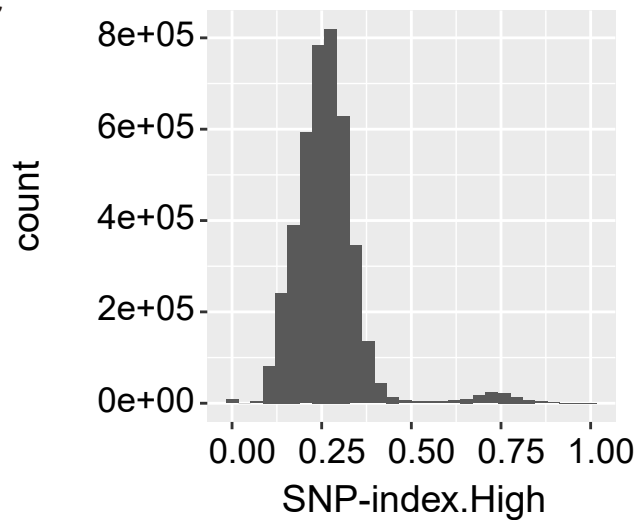

d

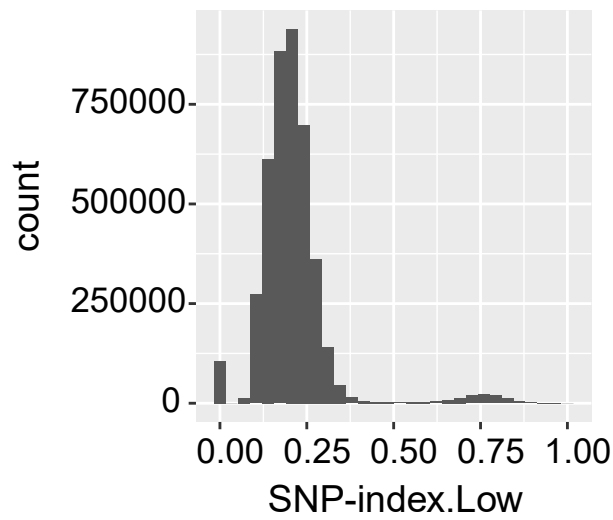

Supplement: Supplementary file 10 — Additional file 10: Figure S5. Title: Frequency distribution of read depth and allele frequency of CSR_L and CSR_H SNPs. Description: (a) Total read depth in two gene pools. (b) Frequency of the reference allele in the two gene pools. C. SNP-index of CSR_H. D. SNP-index of CSR_L. [file 12711_2024_937_MOESM10_ESM.pdf]

0.0 1.3 2.6 3.9 5.2 6.5 7.8 9.1 10.5 11.8 13.1 kb

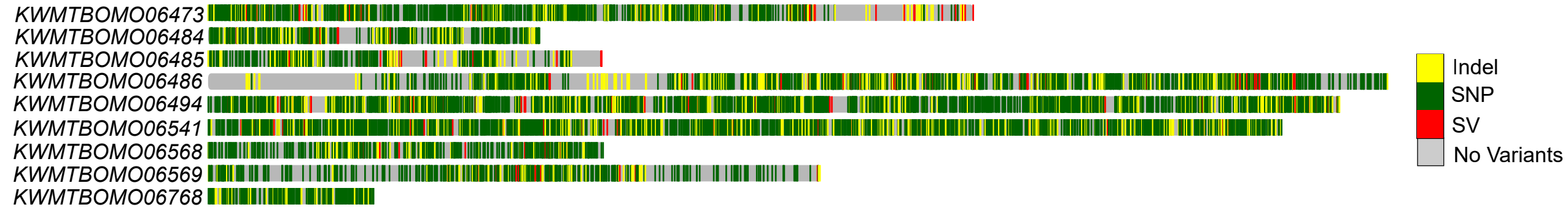

Supplement: Supplementary file 12 — Additional file 12: Figure S6. Title: Detection of genomic sequence variations in the candidate genes for CSR. [file 12711_2024_937_MOESM12_ESM.pdf]

# KWMTBOMO06568 (*BmCdt1*)

5' UTR

Improved

Local

Wild

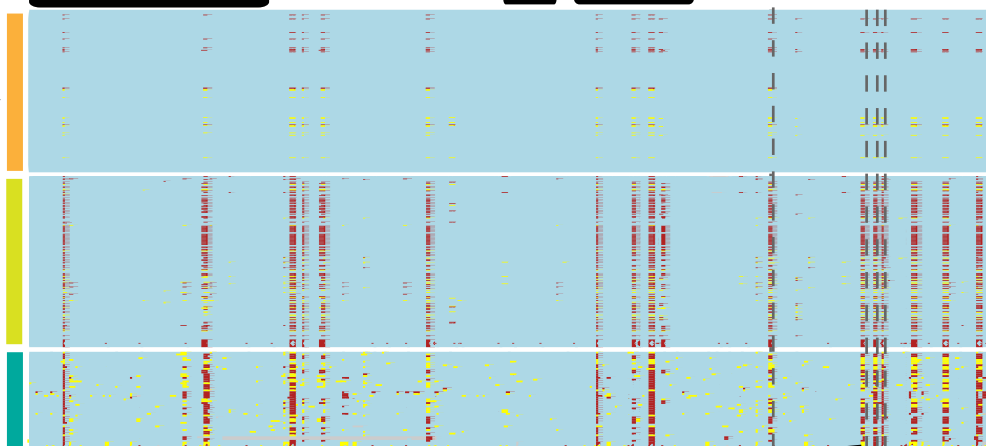

| SNP | -279 | -416 | -467 | -473 | -481 |
|-----|------|------|------|------|------|
| Ref | T    | C    | A    | T    | A    |
| Alt | A    | A    | G    | C    | G    |

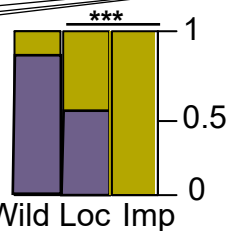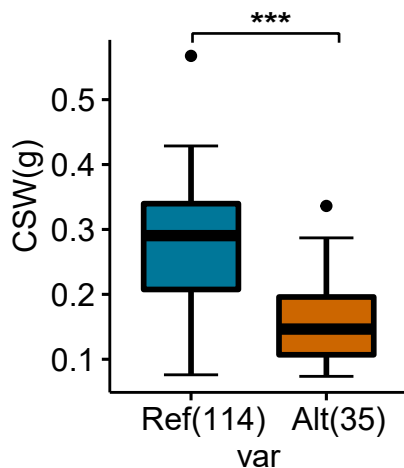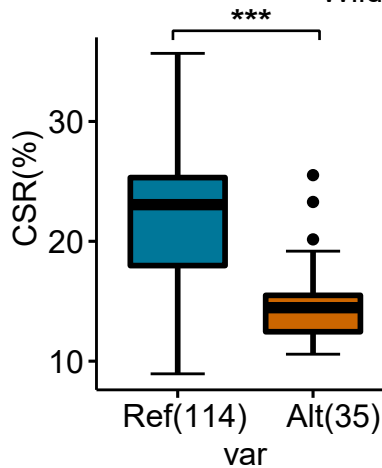

Supplement: Supplementary file 15 — Additional file 15: Figure S7. Title: Haplotype in the 5' UTR of BmCdt1, with the two genotypes that are associated with CSR and CSW between Local and Improved silkworm populations. [file 12711_2024_937_MOESM15_ESM.pdf]

a

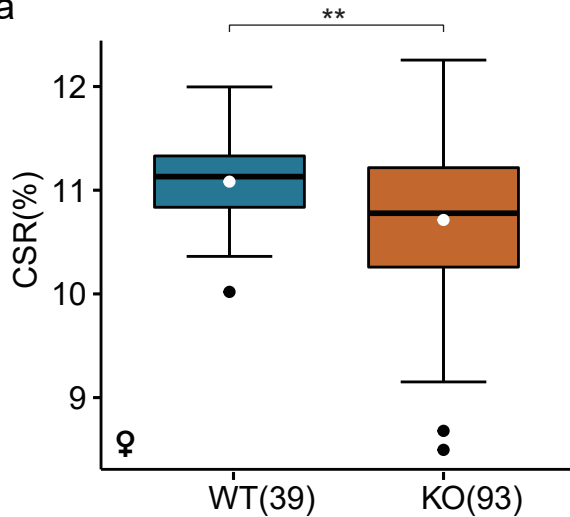

b

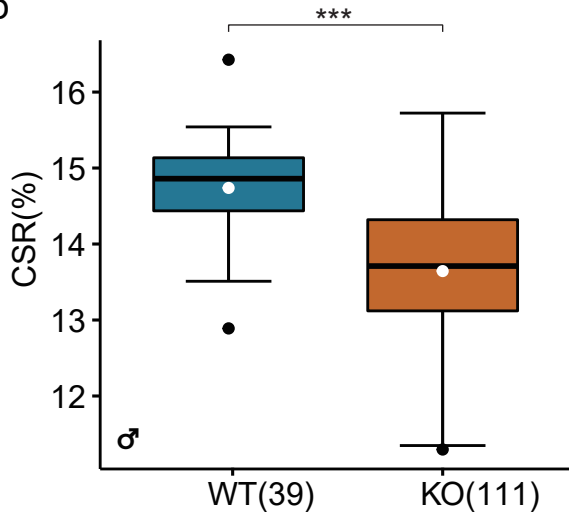

Supplement: Supplementary file 16 — Additional file 16: Figure S8. Title: Phenotypic investigation of CSR in females (left) and males (right) of the wild-type (WT) and BmDnmt2-KO (KO) lines. [file 12711_2024_937_MOESM16_ESM.pdf]
