# Supplementary material for: QTL analysis to identify genes involved in the trade-off between silk protein synthesis and larva-pupa transition in silkworms
Source: Genet Sel Evol. 2024 Sep 30;56:68. doi: 10.1186/s12711-024-00937-z (PMC11440889; doi:10.1186/s12711-024-00937-z)
Supplement: Supplementary file 3 — Additional file 3: Figure S2. Title: Detailed procedure for the analysis of selective sweeps and the signatures of JPN-I Improvement. Description: a. Detailed procedure for the analysis of selective sweeps. b. π of JPN-I; FST and ROD between local and JPN-I silkworm groups in the QTL region on Chr11. [file 12711_2024_937_MOESM3_ESM.pdf]

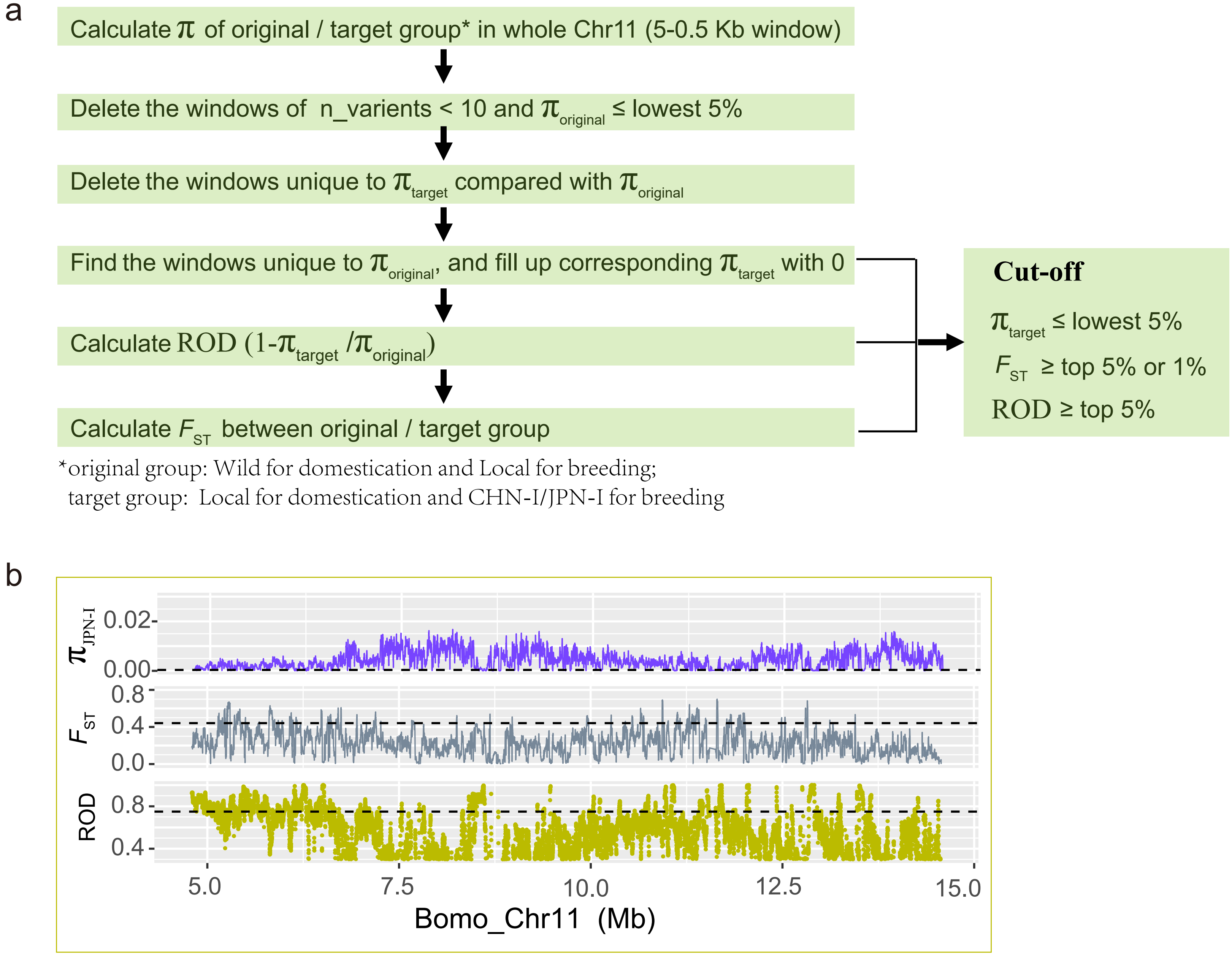
a
Calculate π of original / target group* in whole Chr11 (5-0.5 Kb window)
Delete the windows of n_varients < 10 and π_original ≤ lowest 5%
Delete the windows unique to π_target compared with π_original
Find the windows unique to π_original, and fill up corresponding π_target with 0
Calculate ROD (1-π_target /π_original)
Calculate F_ST between original / target group
Cut-off
π_target ≤ lowest 5%
F_ST ≥ top 5% or 1%
ROD ≥ top 5%
*original group: Wild for domestication and Local for breeding;
target group: Local for domestication and CHN-I/JPN-I for breeding
b
π_JPN-I
0.02
0.00
F_ST
0.8
0.4
0.0
ROD
0.8
0.4
5.0
7.5
10.0
12.5
15.0
Bomo_Chr11 (Mb)
