# Supplementary material for: QTL analysis to identify genes involved in the trade-off between silk protein synthesis and larva-pupa transition in silkworms
Source: Genet Sel Evol. 2024 Sep 30;56:68. doi: 10.1186/s12711-024-00937-z (PMC11440889; doi:10.1186/s12711-024-00937-z)
Supplement: Supplementary file 5 — Additional file 5: Figure S3. Title: The flowchart of screening and sequence detection of homozygous knockout lines. [file 12711_2024_937_MOESM5_ESM.pdf]

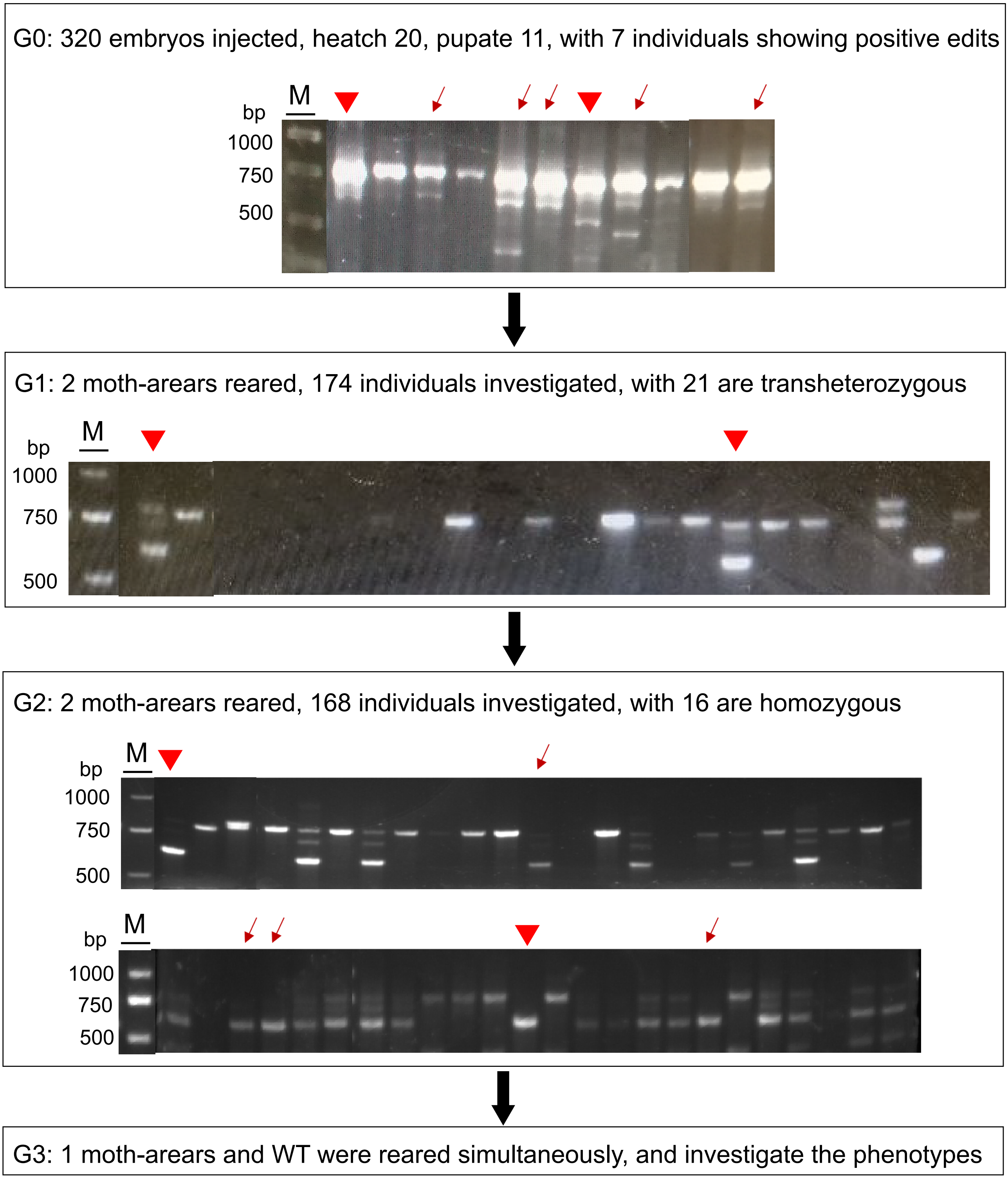
G0: 320 embryos injected, heatch 20, pupate 11, with 7 individuals showing positive edits
M
bp
1000
750
500
G1: 2 moth-arears reared, 174 individuals investigated, with 21 are transheterozygous
M
bp
1000
750
500
G2: 2 moth-arears reared, 168 individuals investigated, with 16 are homozygous
M
bp
1000
750
500
M
bp
1000
750
500
G3: 1 moth-arears and WT were reared simultaneously, and investigate the phenotypes
